# Case report of hepatic artery dissection secondary to hepatic artery pseudoaneurysm after living donor liver transplantation

**DOI:** 10.1186/s12876-016-0458-8

**Published:** 2016-04-01

**Authors:** Lin Ma, Kefei Chen, Qiang Lu, Wenwu Ling, Yan Luo

**Affiliations:** Department of Ultrasound, West China Hospital of Sichuan University, 37 Guoxue Lane, Chengdu, Sichuan Province 610041 China; Department of liver and Vascular Surgery, West China Hospital of Sichuan University, Chengdu, Sichuan Province China

**Keywords:** Hepatic artery pseudoaneurysm, Dissection, Living-donor liver transplantation, Ultrasound, Contrast-enhanced ultrasound

## Abstract

**Background:**

Hepatic artery pseudoaneurysm (HAP) and Hepatic artery dissection are rare vascular complications after living donor liver transplantation (LDLT), which may lead to graft loss and death of the recipients. Conventional gray-scale and Doppler ultrasound, as well as contrast-enhanced ultrasound (CEUS), play important roles in identifying vascular complications in the early postoperative period and during follow-up. We report a case of hepatic artery dissection secondary to HAP after LDLT, which was diagnosed and followed for one year by ultrasound. To the best of our knowledge, few studies have reported similar cases after liver transplantation in the English literature.

**Case presentation:**

A 43-year-old man underwent right-lobe LDLT for treatment of a severe acute hepatitis B infection and was followed up with ultrasound examinations for one year. Conventional gray-scale and Doppler ultrasound combined with contrast-enhanced ultrasound (CEUS) accurately revealed the occurrence of HA dissection secondary to HAP and accompanied by thrombosis and collateral circulation, as well as secondary biliary complications, which provided a prompt diagnosis and guidance for the treatment.

**Conclusion:**

Our case suggests that ultrasound can help detect hepatic artery pseudoaneurysm and dissection, as well as secondary biliary lesions after LDLT in an accurate and timely manner and provide useful information for the treatment chosen. CEUS shows potential as an important complementary technique to gray-scale and Doppler ultrasound.

## Background

Hepatic artery (HA) complications, including hepatic artery thrombosis (HAT), hepatic artery stenosis (HAS), pseudoaneurysm and dissection, are some of the most severe complications after living donor liver transplantation (LDLT), which may lead to graft loss and the recipient’s death [1–8]. HAT and HAS are relatively common arterial complications, with a reported incidence of 3–5 % and 5–11 %, respectively [1–5]. Hepatic artery pseudoaneurysm (HAP) is reported in 0.4–0.5 % of transplant recipients, and HA dissection is rarer [6–8]. The early detection and timely treatment of HA complications are critical for the survival of the graft and recipients.

Imaging techniques play decisive roles in the diagnosis of HA complications. Angiography is the traditional gold standard, but it is invasive; Computed tomography (CT) has radiation as a side effect, and magnetic resonance imaging (MRI) is more expensive; Moreover, these techniques are not available at the bedside for some severe patients in the intensive care unit. As a non-invasive, cost-effective and non-radioactive method with bedside availability, ultrasound serves as a first-line imaging technique to identify vascular complications in the early postoperative period and long-term follow-up [9–14]. Contrast-enhanced ultrasound (CEUS) has been applied gradually in recent years and facilitates visualization of blood vessels by providing real time angiographic-like images with a high diagnostic efficiency [2, 3, 13].

Very few studies have reported HAP or HA dissection [6–8, 15–17]. In this paper, we report a case of HA dissection secondary to HAP and accompanied by thrombosis and collateral circulation as well as secondary biliary complications after LDLT, which was diagnosed and followed for one year by ultrasound. This case report could improve our understanding of the application of ultrasound, especially CEUS, in the diagnosis of rare HA complications after LDLT.

## Case presentation

A 43-year-old male patient suffered from a severe acute hepatitis B infection and liver failure and underwent LDLT with a right lobe graft. The hepatic arteries and portal veins of the donor and recipient, respectively, were anastomosed end-to-end and bile duct reconstruction was performed with a duct-to-duct anastomosis. Intraoperative ultrasound showed no abnormalities in the hepatic artery, portal vein, hepatic vein or bile duct.

After LDLT, the patient received immunosuppression therapy using tacrolimus and metacortandracin. Ultrasonography was performed as a follow-up technique by two experienced ultrasound physicians with more than 5 years of experience in liver transplantation. Conventional gray-scale and Doppler ultrasound was performed every day during the first two weeks after the transplantation, and afterwards, the time and interval of the ultrasound examination depended on the laboratory results and clinical conditions. Contrast-enhanced ultrasound (CEUS) was performed whenever needed.

Within the first 50 days, ultrasound revealed a mild right pleural effusion and ascites, but no significant abnormalities of the HA, portal vein or hepatic vein were observed. Unfortunately, the patient developed acute rejection, accompanied by malnutrition, with abnormal liver function test results and a high total bilirubin level. Therefore, anti-rejection therapy by adjusting the concentration of immunosuppressive drugs was performed in the intensive care unit.

**Fig. 1:**
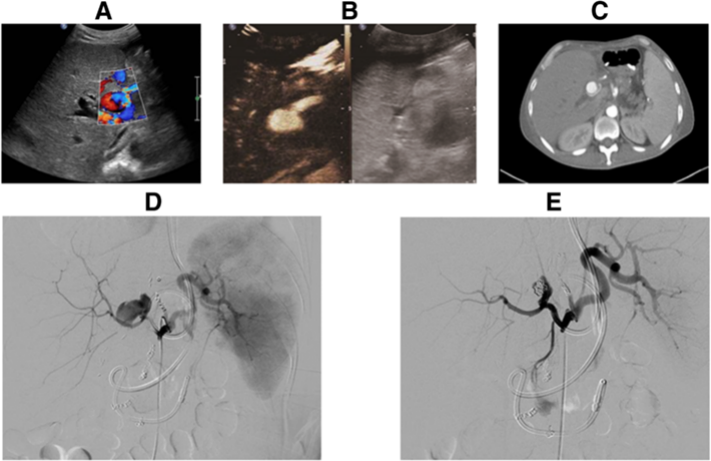
HAP was identified by (**a**) Color Doppler ultrasound, **b** CEUS and **c** Contrast-enhanced CT (*arrow*), and confirmed by (**d**) DSA. **e** The patient underwent coil embolization combined with thrombin injections, and the lumen of the pseudoaneurysm was totally occluded, which was confirmed by DSA

On the 51^st^ postoperative day, the patient developed hematemesis, melena and high fever. Laboratory tests revealed moderate anemia (red blood cell count, 2.4 × 10^12^/l; hemoglobin, 73 g/l). An emergent ultrasound examination was performed, and a homogeneous cystic lesion with a size of 3 × 2 cm was detected in the hilus hepatis, which showed the characteristic red and blue arterial flow pattern on the color Doppler flow image (Fig. 1a). Therefore, a pseudoaneurysm involving the HA allograft was suspected. CEUS was performed immediately at the beside, which further confirmed the diagnosis by demonstrating contrast enhancement in the focal lesion during the arterial phase due to microbubble leakage from the HA (Fig. 1b). Moreover, graft necrosis and abscess did not appear within the hepatic parenchyma on CEUS. Contrast-enhanced computed tomography (Fig. 1c) and digital subtraction angiography (DSA) showed similar findings (Fig. 1d). Interventional stent placement was attempted immediately, but it failed because of the tortuosity of the HA. Subsequently, the patient underwent coil embolization of the HAP combined with thrombin injections. After the embolization, the pseudoaneurysm lumen was totally occluded, as confirmed by DSA (Fig. 1e).

**Fig. 2:**
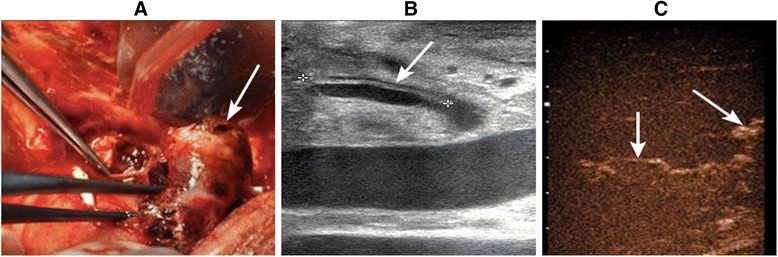
**a** A blood clot that was 4 × 3 cm in size was found in the hilus hepatis during urgent exploratory laparotomy (*arrow*). **b** Hepatic artery dissection was identified by gray-scale ultrasound. **c** Intraoperative CEUS revealed the opening of collateral circulation

Two days later, the patient suddenly had massive rebleeding, and his blood pressure dropped rapidly. These symptoms were strongly indicative of rupture of the HAP. Urgent exploratory laparotomy was performed immediately, and a blood clot that was 4 × 3 cm in size was discovered in the hilus hepatis (Fig. 2a). These findings were accompanied by bile leakage from the common bile duct. Moreover, intraoperative gray-scale ultrasound identified a 2.12-cm peel of the inner membrane of the HA (Fig. 2b), which indicated the occurrence of HA dissection. With this condition, it was almost impossible to perform a surgical repair successfully. Hence, resection of the pseudoaneurysm and HA dissection were performed, followed by arterial reconstruction with interposition bypass via the saphenous vein between the donor right hepatic artery and recipient common hepatic artery. After the reconstructed vessel was reopened, hepatic arterial signals in the hilus hepatis could be detected by intraoperative Doppler ultrasound, but intrahepatic arterial signals could not be detected, which suggested thrombosis of the intrahepatic HA. Attempts at taking out the thrombosis to regain arterial flow failed. However, slender branches of the HA were visualized within the hepatic parenchyma by intraoperative CEUS (Fig. 2c), which indicated the opening of collateral circulation. Taking into account the formation of collateral arterial circulation and the possibility of bleeding because of weakness of the saphenous vein, hepatic artery ligation (HAL) was finally decided on in order to avoid rebleeding from the HA, which had multiple lesions.

**Fig. 3:**
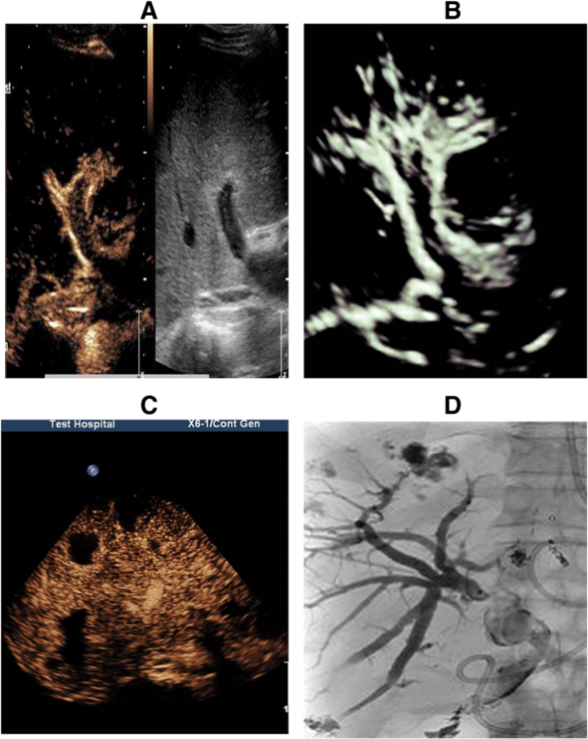
**a** and (**b**) Collateral circulation was observed on both CEUS and 3D-CEUS. **c** and (**d**) Bilomas were observed on CEUS and PTCD

After the HAL, the patient was hemodynamically stable and did not have further bleeding. The follow-up examinations revealed an absence of blood flow in the HA on conventional ultrasound scans, as well as collateral circulation of the HA on both CEUS and 3D-CEUS scans (Fig. 3a and b).

Biliary complications arising from hepatic artery ischemia were also detected. At the first postoperative month, gray-scale ultrasound revealed mild dilation of the intrahepatic bile duct and thickening of the wall of the extrahepatic bile duct, and later, multiple cystic-solid mixed lesions with a maximum diameter of 4 cm in the hepatic parenchyma were observed. CEUS showed no enhancement in these lesions (Fig. 3c). Then, percutaneous transhepatic cholangial drainage (PTCD) was performed to remove the obstruction in the bile duct, and during the procedure, the lesions were confirmed as bilomas (Fig. 3d), which became smaller and disappeared 5 months later. Additionally, at the fourth postoperative month, biliary cast formation was observed.

Unfortunately, the patient developed severe bacterial and fungal infections involving the lung, biliary and intestinal tracts, which may have been caused by the use of immunosuppressants and metacortandracin, bile leakage, malnutrition and iatrogenic factors. Although he was hospitalized and treated for one year, multiple organ failure occurred, leading to death.

## Discussion

This case is significant because, to the best of our knowledge, HA dissection secondary to HAP accompanied by thrombosis and collateral circulation after LDLT has been rarely reported in the English literature. Although uncommon, HAP and HA dissection have a high morbidity and mortality, and early diagnosis and treatment are important [6–8, 15–17].

HAP can be classified as intrahepatic or extrahepatic according to its location. Intrahepatic pseudoaneurysms may result from previous procedures, such as liver biopsy and percutaneous transhepatic biliary drainage, whereas extrahepatic pseudoaneurysms are commonly associated with localized infection or anastomotic complications [6, 15–17]. In our case, HAP was most likely the result of an infected injury to the HA intima layer caused by bile leakage. We presumed that bile leakage might have occurred in the early postoperative period, but it was not initially recognized because of the misdiagnosis as ascites of the sub-hepatic space; hence ultrasound-guided effusion punctures should be performed to assess the properties of peritoneal effusions, which may be beneficial for being vigilant on the initial bleeder. Moreover, the aneurysm arose at the HA close to the anastomosis, and thus the anastomosis might be another factor. The clinical manifestations of HAP are diverse and include fever, bile leak, hepatic dysfunction, abdominal pain, hematemesis, melena, anemia, hypotension and jaundice, some of which are asymptomatic and detected incidentally during routine examination. Intra-abdominal or gastrointestinal hemorrhages from rupture are the most severe presentations of HAP and are life-threatening and the main cause of postoperative death [15–17]. Early diagnosis and treatment are critical for graft salvage. DSA is the most reliable imaging modality in the evaluation of an aneurysm, but it was not considered as an initial procedure because of its invasiveness and complexity. Ultrasound has been considered the primary noninvasive imaging technique to detect HAP, and the ability to perform this examination at the patient’s bedside and the absence of radiation hazards make it an ideal first-line examination. CEUS can significantly improve the visualization of blood vessels, and it can be a superior technique for evaluating pseudoaneurysms because of its high sensitivity and real-time scanning [2, 9–14]. In our case, both Doppler ultrasound and a CEUS scan accurately revealed HAP based on its typical manifestation. Moreover, CEUS can be used to access perfusion in the liver parenchyma, facilitating identifying graft necrosis and abscess. It is often difficult to visualize blood flow in small arterial branches by conventional gray-scale and Doppler ultrasound, which is considered as one of the main limitations [18]. In our case, slender arterial branches in the hepatic parenchyma were visualized only with CEUS, which indicated the opening of collateral circulation and provided useful information for the treatment chosen Thus, CEUS may be an important complementary technique for conventional gray-scale and Doppler ultrasound; it can improve the effectiveness of ultrasound in the diagnosis of HA diseases.

HA dissection is recognized as a rare complication of LDLT. To the best of our knowledge, 23 cases have been reported in the English literature, and only one has been documented in a case of liver transplantation [7, 8]. Asonuma K et al. [7] reported intimal dissection of the recipient’s HA with no thrombosis in 2004; this occurred immediately after the transplantation and was detected by Doppler ultrasonography, but the cause was unknown. In 71 % of reported cases, HA dissections were symptomatic, typically presenting with pain in the epigastrium and right hypochondrium. Severe secondary complications, such as HA thrombosis and subsequent graft loss, may occur and the rupture with shock could always rapidly lead to death [19]. The causes of HA dissection may include atherosclerosis, trauma, fibromuscular dysplasia, connective tissue disease and iatrogenic factors [20]. In our case, the patient underwent attempted HA angioplasty with stent placement and arterial embolization, which may have caused mechanical trauma to the intimal layer of the arterial wall or damage to the intima and made the vessel fragile, resulting in intimal dissection of the HA. Conventional gray-scale and Doppler ultrasound are effective methods for the diagnosis of HA dissection and can be the first-line imaging examination because they are good for identifying the separation of the intimae, blood flow separation and intraluminal thrombosis, as shown in our case.

Bile ducts are supplied by the hepatic artery only, and the biliary epithelium is more sensitive to ischemic injury than hepatocytes [21]. Ischemia resulting from HA diseases initially affects the bile ducts, which may lead to biliary necrosis, cast formation, abscesses, non-anastomotic bile leak and bilomas [21, 22]. In our case, the bilomas could be clearly observed by conventional ultrasound and CEUS and gradually disappeared due to blood supply to the collateral arteries. Thickened bile duct walls and biliary casts were also well demonstrated. Therefore, conventional ultrasound and CEUS can be considered efficient imaging methods for depicting the bile duct wall, biliary luminal and biloma development.

## Conclusion

HAP and HA dissection are rare complications of LDLT and can result in severe consequences. The findings from our case suggest that ultrasound can help detect hepatic artery pseudoaneurysm and dissection, as well as secondary biliary lesions after LDLT in an accurate and timely manner and provide useful information for the treatment chosen. CEUS has potential as an important complementary approach along with conventional gray-scale and Doppler ultrasound.

## Consent

Written informed consent was obtained from the patient’s family for publication of this Case Report and any accompanying images. A copy of the written consent is available for review by the Editor-in-Chief of this journal.

## Abbreviations

CEUS: contrast-enhanced ultrasound
CT: computed tomography
DSA: digital subtraction angiography
HA: hepatic artery
HAL: hepatic artery ligation
HAP: hepatic artery pseudoaneurysm
HAS: hepatic artery stenosis
HAT: hepatic artery thrombosis
LDLT: living donor liver transplantation
MRI: magnetic resonance imaging
PTCD: percutaneous transhepatic cholangial drainage
